# Occult Bartonella Quintana Infective Endocarditis Incidentally Diagnosed During Pre-Transcatheter Aortic Valve Implantation (TAVI) Workup in a Patient Without Classical Risk Factors

**DOI:** 10.7759/cureus.115582

**Published:** 2026-09-01

**Authors:** Nouhaila Bentama, Malak Alaoui Yazidi, Jalal Tagueniti, Brahim Meftout

**Affiliations:** 1 Cardiology, Ibn Sina Hospital, Mohammed V University, Rabat, MAR; 2 Medicine, Centre Hospitalier Sud Francilien, Corbeil-Essonnes, FRA; 3 Cardiology, Centre Hospitalier Sud Francilien, Corbeil-Essonnes, FRA

**Keywords:** 2023 duke-iscvid criteria, aortic root abscess, bartonella, blood culture-negative infective endocarditis, cerebral mycotic aneurysm, infective endocarditis, transcatheter aortic valve implantation (tavi)

## Abstract

*Bartonella* infective endocarditis (IE) is a rare cause of initially blood culture-negative IE, classically associated with cat flea exposure in *Bartonella henselae* infection or body lice infestation among individuals experiencing homelessness or alcoholism in *Bartonella quintana* infection. We report the case of an 84-year-old man with severe calcific aortic stenosis who was undergoing evaluation for pre-transcatheter aortic valve implantation (TAVI). Cardiac computed tomography (CT) unexpectedly revealed a peri-annular cavity consistent with a drained or healed peri-annular abscess, despite the absence of fever or other clinical features of IE. Initial blood cultures were negative, and TAVI was planned following multidisciplinary discussion, pending serological results. However, a subsequently reported high-titer *Bartonella *serology prompted readmission, and TAVI was ultimately deferred after the diagnosis was confirmed. Prolonged incubation of blood cultures subsequently identified *B. quintana*, while brain magnetic resonance imaging (MRI) revealed a lesion suspicious for an intracranial infectious aneurysm. Definite IE was established according to the 2023 Duke-International Society for Cardiovascular Infectious Diseases (Duke-ISCVID) criteria based on two major criteria: high-titer *Bartonella *serology and imaging evidence of a peri-annular abscess. No classical epidemiological risk factors for *Bartonella *infection were identified. Despite targeted antimicrobial therapy, the patient experienced progressive clinical deterioration, and multiple comorbidities precluded surgical intervention. He ultimately died of multiorgan failure. This case highlights the diagnostic challenge of occult *B. quintana* IE incidentally identified during pre-TAVI evaluation, including in patients without classical epidemiological risk factors or overt manifestations of infection. It also emphasizes that unexpected peri-annular abnormalities detected during pre-TAVI evaluation should prompt a thorough investigation for IE, even in clinically unsuspected cases, with direct implications for procedural planning.

## Introduction

*Bartonella *species are among the leading causes of blood culture-negative infective endocarditis (BCNIE), yet their diagnosis remains challenging because of their nonspecific clinical presentation and fastidious microbiological characteristics. *Bartonella *accounts for approximately 3% of all cases of infective endocarditis (IE) [1], while recent European cohorts have reported a prevalence of approximately 0.3% among all IE cases and 2% among BCNIE cases [2]. More than 95% of *Bartonella *endocarditis cases are caused by *Bartonella quintana* and *Bartonella henselae*. *B. quintana* infection is classically associated with body lice infestation and socioeconomically disadvantaged populations, whereas *B. henselae* infection is typically linked to cat exposure through scratches or infected fleas [1,3]. Because conventional blood cultures are frequently negative, diagnosis primarily relies on serological testing and molecular techniques [1].

The 2023 update of the Duke-International Society for Cardiovascular Infectious Diseases (Duke-ISCVID) diagnostic criteria represented a major advance in the diagnosis of IE by recognizing *Bartonella *serology as a major microbiological criterion and expanding the role of multimodality imaging, including cardiac computed tomography (CT) and 18F-fluorodeoxyglucose positron emission tomography/computed tomography (18F-FDG PET/CT) [4]. These updates have improved the diagnostic sensitivity for BCNIE and have been incorporated into the 2023 European Society of Cardiology guidelines for the management of IE [4,5].

We report the case of an elderly patient in whom *B. quintana* IE was incidentally identified during pre-transcatheter aortic valve implantation (TAVI) evaluation in the absence of clinical suspicion. This case illustrates how unexpected peri-annular findings on pre-TAVI cardiac CT can uncover previously unsuspected IE. This distinction is clinically critical, as unrecognized peri-annular infection would contraindicate valve implantation into an infected aortic root. The case therefore highlights the diagnostic contribution of the updated Duke-ISCVID criteria in an initially blood culture-negative presentation, as well as the therapeutic challenges posed by advanced peri-annular complications in a frail elderly patient.

## Case presentation

An 84-year-old man was referred for evaluation of severe symptomatic calcific aortic stenosis following his first episode of acute decompensated heart failure. His medical history included hypertension, recently diagnosed parkinsonism, and immune thrombocytopenia associated with an underlying lymphoproliferative disorder. He reported no history of cat exposure, body lice infestation, homelessness, alcoholism, or any other recognized epidemiological risk factors for *Bartonella *infection.

Transthoracic echocardiography (TTE) demonstrated severe calcific aortic stenosis, with a mean transvalvular gradient of 60 mmHg, a peak aortic jet velocity of 4.7 m/s, and an indexed aortic valve area of 0.43 cm²/m² (Figure 1). As part of the routine pre-TAVI workup, cardiac CT unexpectedly revealed a peri-annular cavity at the junction of the non-coronary and right coronary sinuses, consistent with a chronic drained/healed peri-annular abscess cavity. The aortic valve calcium score was 4,016 Agatston units.

**Figure 1:**
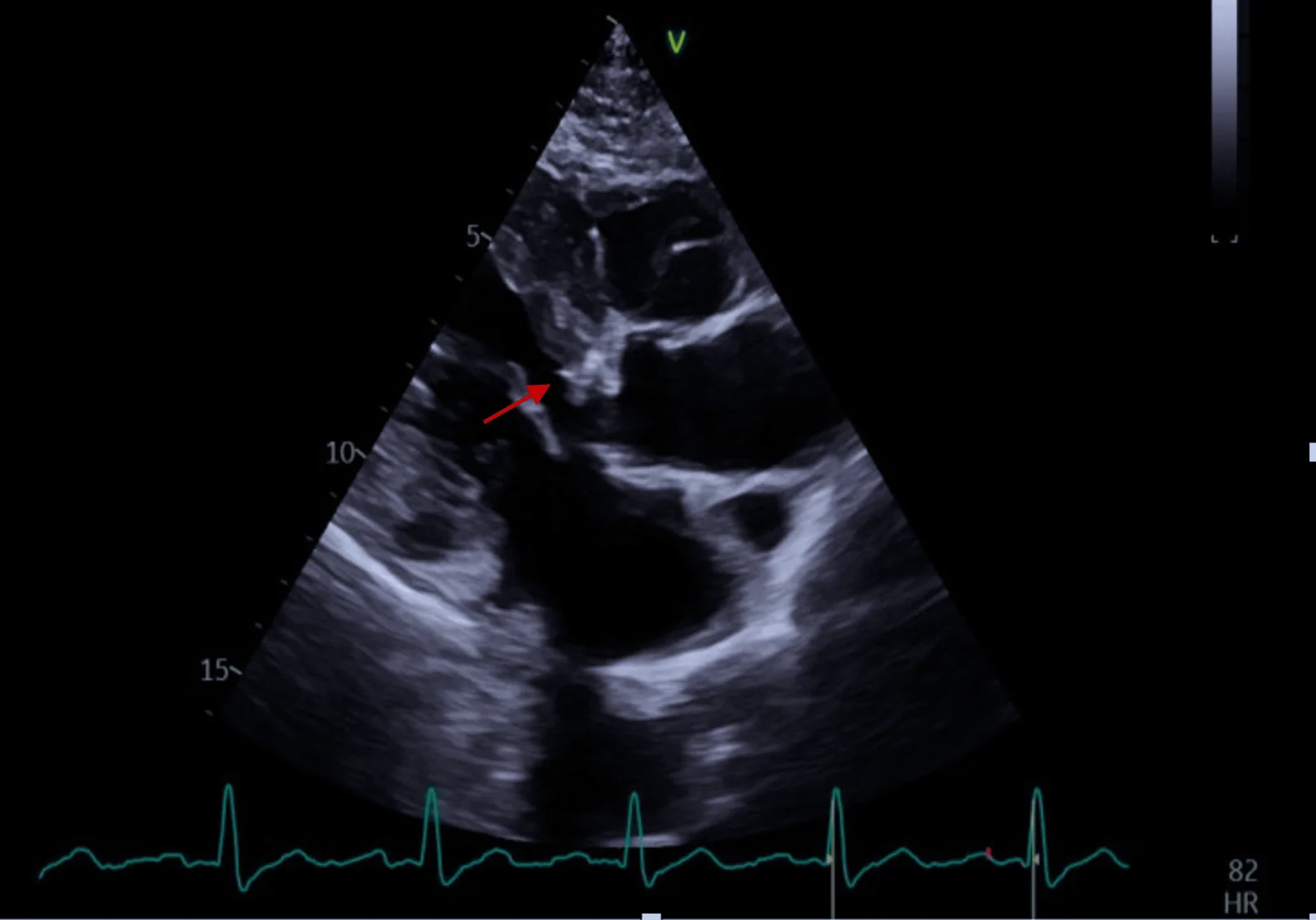
Transthoracic echocardiography (parasternal long-axis view) demonstrating severe calcific aortic stenosis with markedly thickened and heavily calcified aortic valve leaflets

Despite this finding, the patient had no clinical manifestations suggestive of IE. He was afebrile, with only mildly elevated inflammatory markers (C-reactive protein, 51 mg/L) in the setting of recent acute heart failure. Two sets of blood cultures remained negative; however, they had been obtained after empirical antibiotic therapy had been initiated during the patient's previous admission for acute heart failure decompensation, potentially reducing their diagnostic yield (Table 1). The specific antimicrobial agent and treatment duration could not be retrieved from the available medical records. Given the unexpected CT findings, serological testing for *Bartonella*, *Coxiella burnetii*, *Brucella *spp., and *Legionella *spp. was requested.

**Table 1:** Key investigations, results, and clinical significance MALDI-TOF: Matrix-assisted laser desorption/ionization time-of-flight mass spectrometry; ISCVID: International Society for Cardiovascular Infectious Diseases; TAVI: Transcatheter aortic valve implantation; TTE: Transthoracic echocardiography; TEE: Transesophageal echocardiography; 18F-FDG PET/CT: 18F-Fluorodeoxyglucose positron emission tomography/computed tomography.

Investigation	Result	Clinical significance
CRP	51 mg/L	Mild inflammatory syndrome
Blood cultures (initial)	Negative (after prior antibiotics)	Possible false-negative result
*Bartonella henselae* IgG	1/2048 → 1/4096	Major Duke-ISCVID microbiological criterion
*Bartonella *blood culture	*Bartonella quintana *identified by MALDI-TOF	Microbiological confirmation
Cardiac CT	Peri-annular cavity consistent with drained/healed abscess	Major Duke-ISCVID imaging criterion
TTE	Severe calcific aortic stenosis	Indication for TAVI
TEE	Unsuccessful	Poor patient tolerance
Brain MRI	Lesion suspicious for an intracranial infectious aneurysm	Embolic complication
18F-FDG PET/CT	No valvular uptake	Performed after antibiotics

Although the CT findings raised suspicion of prior peri-annular infection, active IE was considered clinically unlikely at that stage, given the absence of fever, the only mildly elevated inflammatory markers, the negative blood cultures, and the lack of echocardiographic vegetation. TAVI was therefore initially planned; however, the procedure was formally deferred pending the results of the requested serological workup, and the patient was discharged with the TAVI indication provisionally maintained but not scheduled to proceed until microbiological clarification. Following the subsequent diagnosis of definite IE, TAVI was ultimately not performed.

Three weeks later, the patient was readmitted after *Bartonella *serology returned strongly positive (*B. henselae *IgG titer 1/2048; IgM negative). He reported an unintentional 10-kg weight loss over the preceding two months but denied fever, chills, night sweats, or any history of cat scratches or bites. Physical examination revealed a systolic ejection murmur radiating to the carotid arteries, bilateral lower-limb edema, a left lateral malleolar ulcer, and poor oral hygiene, without peripheral stigmata of IE.

Further microbiological investigations supported the diagnosis of *Bartonella *IE. A blood culture obtained on admission became positive after prolonged incubation (day 8), with subsequent identification of *B. quintana *by matrix-assisted laser desorption/ionization time-of-flight mass spectrometry (MALDI-TOF); the specific MALDI-TOF platform and confirmatory molecular testing (PCR/16S rRNA sequencing) were not performed. Repeat *Bartonella *IgG serology (against *B. henselae*) demonstrated a further increase to 1/4096; the specific serological technique used could not be confirmed from the available records, and serology against *B. quintana *was not separately performed. *Chlamydia *serology was weakly positive, most likely reflecting the well-described serological cross-reactivity between *Bartonella *and *Chlamydia *species [6]. Two additional blood culture sets yielded a slow-growing Gram-negative bacillus after 13 days of incubation; pending definitive characterization, ceftriaxone was added to ensure appropriate antimicrobial coverage. Despite this, definitive species identification could not subsequently be obtained, and this isolate remains unresolved as either a possible HACEK group (*Haemophilus *species, *Aggregatibacter actinomycetemcomitans*, *Cardiobacterium hominis*, *Eikenella corrodens*, and *Kingella kingae*) co-infection or contamination, representing a limitation of this report.

On retrospective review by the multidisciplinary endocarditis team, TTE was considered compatible with a drained/healed peri-annular abscess cavity involving the mitral-aortic intervalvular fibrosa, although no vegetation was identified on either the initial or the repeat examination; the final characterization of the lesion therefore relied predominantly on cardiac CT. Transesophageal echocardiography (TEE) was attempted twice but could not be completed because of poor patient tolerance. 18F-fluorodeoxyglucose positron emission tomography/computed tomography (18F-FDG PET/CT), performed after six days of antimicrobial therapy, showed no abnormal valvular uptake, a finding interpreted cautiously because prior antibiotic treatment may reduce PET sensitivity. Incidentally, focal duodenal hypermetabolism was also observed. Brain magnetic resonance imaging (MRI) revealed diffuse pachymeningeal enhancement and a small left occipital subcortical T2-weighted hypointense lesion with adjacent leptomeningeal enhancement. As no dedicated vascular imaging (MR angiography, CT angiography, or digital subtraction angiography) was performed to confirm an aneurysm, this finding is more accurately described as a lesion suspicious for an intracranial infectious aneurysm rather than a confirmed mycotic aneurysm. Following multidisciplinary discussion with the interventional neuroradiology team, conservative management was favored because there was no evidence of intracranial hemorrhage and neither therapeutic anticoagulation nor cardiac surgery was planned.

Based on these findings, the diagnosis of definite IE was established according to the 2023 Duke-ISCVID diagnostic criteria [4]. Two major criteria were fulfilled: a *Bartonella *IgG titer of 1/2048 (rising to 1/4096), exceeding the 1/800 threshold recognized as a major microbiological criterion, and cardiac CT demonstrating a peri-annular cavity at the aortic root consistent with a drained/healed peri-annular abscess, fulfilling a major imaging criterion. The lesion suspicious for an intracranial infectious aneurysm and the negative 18F-FDG PET/CT (interpreted cautiously given six days of prior antimicrobial therapy) were considered additional supportive, non-major findings.

Doxycycline and rifampicin were selected as the primary antimicrobial regimen for *Bartonella *IE, in line with current recommendations [7,8]. However, gentamicin - the guideline-recommended companion agent to doxycycline [7] - was considered unsafe given the patient's advanced age and pre-existing renal impairment, which substantially increased the risk of aminoglycoside-induced nephrotoxicity. Rifampicin was discontinued after 14 days because of grade 2 peptic esophagitis, antral gastritis, and a deep duodenal bulbar ulcer attributed to antimicrobial-related gastrointestinal toxicity, following consultation with a national reference center; doxycycline was continued for a total treatment duration of six weeks. Ceftriaxone was administered concomitantly while awaiting definitive characterization of the slow-growing Gram-negative bacillus isolated from the additional blood cultures.

The hospital course was further complicated by severe gastrointestinal toxicity related to antimicrobial therapy, newly diagnosed cirrhosis, and multifactorial acute kidney injury superimposed on chronic kidney disease. Despite repeated multidisciplinary discussions, surgical aortic valve replacement was not considered appropriate given progressive clinical deterioration, severe sarcopenia, and multiple comorbidities. The patient was referred to a tertiary center for further evaluation by the endocarditis team; both cardiac surgery and intensive care management were ultimately not offered, as the multidisciplinary team judged the operative and procedural risk to be prohibitive given the patient's overall clinical trajectory. His condition progressively deteriorated, with worsening consciousness, anuria, and multiorgan failure. Following discussion with the patient and his family, the goals of care were transitioned to comfort-focused care. Despite appropriate antimicrobial therapy, the patient developed progressive multiorgan failure and died approximately one month after the initial diagnosis (Table 2).

**Table 2:** Clinical timeline ISCVID: International Society for Cardiovascular Infectious Diseases; IE: Infective endocarditis; TAVI: Transcatheter aortic valve implantation.

Time	Event
Initial admission	Acute heart failure and severe aortic stenosis diagnosed
Pre-TAVI CT	Peri-annular cavity suspicious for a chronic abscess incidentally detected
Initial blood cultures	Negative after prior antibiotics
Discharge	*Bartonella *serology requested
Three weeks later	Readmission after positive serology
Repeat investigations	*Bartonella quintana *identified; MRI showed a lesion suspicious for an intracranial infectious aneurysm
Endocarditis team	Definite IE diagnosed according to Duke-ISCVID 2023
Treatment	Doxycycline + rifampicin
Outcome	Progressive multiorgan failure and death

## Discussion

This case highlights three key clinical challenges associated with *Bartonella* IE: the diagnostic difficulty in the absence of recognized epidemiological risk factors, the contribution of the 2023 Duke-ISCVID criteria in establishing the diagnosis of an initially BCNIE, and the therapeutic challenges encountered in a frail elderly patient with advanced peri-annular complications.

An atypical epidemiological profile

Unlike the classical epidemiological profile of *Bartonella *endocarditis, in which *B. quintana *infection is associated with homelessness, alcoholism, or body lice infestation and *B. henselae* infection is associated with cat exposure [1,3], our patient had none of these recognized risk factors, and no definite portal of entry was identified despite detailed epidemiological assessment. Similar atypical presentations have recently been reported, including cases of *B. quintana* IE occurring in patients without any identifiable epidemiological exposure [9]. Therefore, the absence of classical risk factors should not discourage clinicians from considering *Bartonella *infection in patients presenting with BCNIE, particularly when destructive valvular lesions or peri-annular complications are identified.

Poor oral hygiene was the only potential portal of entry identified in our patient; however, its role in *Bartonella *infection remains entirely speculative. Although no causal relationship has been established, this observation highlights that the source of *Bartonella *infection may remain unidentified despite comprehensive clinical investigation.

Microbiological diagnosis and serological cross-reactivity

The coexistence of positive *B. henselae *serology and blood culture isolation of *B. quintana* in our patient highlights a well-recognized limitation of serological diagnosis. Indirect immunofluorescence assays and enzyme immunoassays exhibit substantial cross-reactivity between *B. henselae* and *B. quintana*, preventing reliable species differentiation on the basis of serology alone [10,11]. A similar discrepancy involving positive *B. henselae* serology due to cross-reactivity was recently reported in a patient with confirmed *B. quintana* IE [9]. In our case, prolonged blood culture incubation followed by MALDI-TOF mass spectrometry enabled definitive identification of the causative species; the specific serological assay used in our patient could not be confirmed from the available records, and *B. quintana*-specific serology was not performed, precluding direct titer comparison between species. Although this distinction has limited therapeutic implications because antimicrobial management is similar for both species [10], it underscores the value of prolonged blood culture incubation and, whenever available, molecular techniques such as PCR for accurate microbiological diagnosis.

Contribution of the 2023 Duke-ISCVID criteria

The 2023 Duke-ISCVID diagnostic criteria represent a major advance in the diagnosis of IE caused by fastidious microorganisms such as *Bartonella*. Unlike the modified Duke criteria published in 2000, which relied predominantly on conventional blood culture results, the updated criteria recognize high-titer *Bartonella *serology as a major microbiological criterion and incorporate multimodality imaging, including cardiac CT and 18F-FDG PET/CT, as major imaging criteria [4]. Subsequent external validation studies have demonstrated improved diagnostic sensitivity compared with both the modified Duke criteria and the 2015 European Society of Cardiology criteria, without compromising specificity [12,13]. In our patient, the negative 18F-FDG PET/CT scan did not exclude the diagnosis, as the examination was performed after six days of antimicrobial therapy, a well-recognized factor that reduces PET sensitivity for IE [5]. This case illustrates the complementary role of multimodality imaging and updated microbiological criteria in establishing the diagnosis in an initially blood culture-negative presentation of IE.

Management of the cerebral mycotic aneurysm

Intracranial infectious aneurysms complicate approximately 2%-10% of cases of IE and are associated with a substantial risk of intracranial hemorrhage and death [14]. The optimal management of unruptured lesions remains controversial. Conservative treatment with appropriate antimicrobial therapy is generally considered the initial strategy, as some aneurysms regress following effective treatment of the underlying infection. However, persistent lesions remain at risk of rupture, emphasizing the importance of close radiological surveillance and multidisciplinary management, particularly before cardiac surgery requiring systemic anticoagulation [15,16]. In our patient, in the absence of dedicated vascular imaging, the left occipital lesion was considered suspicious for an intracranial infectious aneurysm rather than a confirmed diagnosis. Given the absence of intracranial hemorrhage and the lack of an indication for immediate cardiac surgery, a conservative management strategy with close clinical and radiological surveillance was adopted.

Therapeutic challenges in a frail elderly patient

Current European Society of Cardiology guidelines strongly recommend early surgery for IE complicated by peri-annular abscesses [5]. Nevertheless, applying guideline-directed recommendations to frail elderly patients remains challenging. Formal surgical risk scores (EuroSCORE II and STS-PROM) could not be calculated retrospectively because of incomplete availability of required variables in the medical records. Instead, the multidisciplinary endocarditis team's decision against surgery relied on a qualitative assessment integrating advanced age, severe sarcopenia, progressive multiorgan deterioration, newly diagnosed cirrhosis, and acute-on-chronic kidney injury (creatinine 305 µmol/L). This case illustrates the complex balance between guideline-directed management and individualized clinical decision-making in frail elderly patients, in whom the theoretical benefits of surgery may be outweighed by an unacceptable operative risk.

## Conclusions

This case underscores that unexpected peri-annular abnormalities identified during pre-TAVI assessment warrant thorough investigation for IE before proceeding with valve intervention, even in the absence of clinical suspicion. It also illustrates the diagnostic value of the 2023 Duke-ISCVID criteria, which integrate high-titer *Bartonella *serology with multimodality imaging to facilitate the diagnosis of initially blood culture-negative and otherwise elusive cases such as this case, including in patients without recognized epidemiological risk factors. Finally, this case highlights the therapeutic challenges in frail elderly patients with complicated IE, in whom guideline-recommended surgical management may not be feasible despite clear indications.
